# A Specially Designed Multi-Gene Panel Facilitates Genetic Diagnosis in Children with Intrahepatic Cholestasis: Simultaneous Test of Known Large Insertions/Deletions

**DOI:** 10.1371/journal.pone.0164058

**Published:** 2016-10-05

**Authors:** Neng-Li Wang, Yu-Lan Lu, Ping Zhang, Mei-Hong Zhang, Jing-Yu Gong, Yi Lu, Xin-Bao Xie, Yi-Ling Qiu, Yan-Yan Yan, Bing-bing Wu, Jian-She Wang

**Affiliations:** 1 Department of Pediatrics, Jinshan Hospital of Fudan University, Shanghai, China; 2 The Molecular Genetic Diagnosis Center, Shanghai Key Lab of Birth Defects, Pediatrics Research Institute, Children’s Hospital of Fudan University, Shanghai, China; 3 The Center for Pediatric Liver Diseases, Children’s Hospital of Fudan University, Shanghai, China; Oslo Universitetssykehus, NORWAY

## Abstract

**Background and Aims:**

Large indels are commonly identified in patients but are not detectable by routine Sanger sequencing and panel sequencing. We specially designed a multi-gene panel that could simultaneously test known large indels in addition to ordinary variants, and reported the diagnostic yield in patients with intrahepatic cholestasis.

**Methods:**

The panel contains 61 genes associated with cholestasis and 25 known recurrent large indels. The amplicon library was sequenced on Ion PGM system. Sequencing data were analyzed using a routine data analysis protocol and an internal program encoded for large indels test simultaneously. The validation phase was performed using 54 patients with known genetic diagnosis, including 5 with large insertions. At implement phase, 141 patients with intrahepatic cholestasis were evaluated.

**Results:**

At validation phase, 99.6% of the variations identified by Sanger sequencing could be detected by panel sequencing. Following the routine protocol, 99.8% of false positives could be filtered and 98.8% of retained variations were true positives. Large insertions in the 5 patients with known genetic diagnosis could be correctly detected using the internal program. At implementation phase, 96.9% of the retained variations, following the routine protocol, were confirmed to be true. Twenty-nine patients received a potential genetic diagnosis when panel sequencing data were analyzed using the routine protocol. Two additional patients, who were found to harbor large insertions in *SLC25A13*, obtained a potential genetic diagnosis when sequencing data were further analyzed using the internal program. A total of 31 (22.0%) patients obtained a potential genetic diagnosis. Nine different genetic disorders were diagnosed, and citrin deficiency was the commonest.

**Conclusion:**

Specially designed multi-gene panel can correctly detect large indels simultaneously. By using it, we assigned a potential genetic diagnosis to 22.0% of patients with intrahepatic cholestasis.

## Introduction

Cholestasis results from impairment of bile acid biosynthesis, bile secretion and excretion [1]. The etiology is diverse, and includes a range of genetic defects that represent a collection of disorders [2]. The pathophysiology of cholestasis is complex, and depends on the specific genetic defects. Although subtle clinical and biochemical differences exist, these genetic disorders are difficult to be differentiated based on clinical and routine laboratory findings [3–4]. Meanwhile, patients with same genetic defects can present different clinical phenotypes [5–6]. Therefore, genetic tests are extremely important for the establishment of a clear genetic diagnosis, and hence for the initiation of tailored treatment and genetic counseling [3,7].

Quite often, several candidate genes have to be evaluated in clinic practice, because the differential diagnoses are numerous [2]. If the candidate genes are tested by Sanger sequencing, the process is time-consuming and expensive. Multi-gene panel, a time and cost efficient alternative to Sanger sequencing, can screen multiple candidate genes simultaneously [8], and is increasingly used for diagnostic evaluation of patients with intrahepatic cholestasis in children [2,9–10]. However, large insertions/deletions (indels) can neither be detected by routine Sanger sequencing [6,11], nor by routine multi-gene panel sequencing [2,10]. Furthermore, some special large indels, i.e. IVS16ins3kb and IVS4ins6kb in *SLC25A13*, even can’t be detected by copy number variation (CNV) analysis, but have high frequency in patients and contribute substantively to disease burden [12–13].

To facilitate genetic diagnosis, we specially designed a multi-gene panel that could not only sequence the coding exons of 61 cholestasis-related genes, but also test 25 known recurrent large indels (>150bp) in the genes. In this study, we first validated our system using 54 patients with known genetic diagnosis, then evaluated 141 consecutive patients with intrahepatic cholestasis using this panel and reported the diagnostic yield.

## Methods

### Multi-Gene Panel Design

**Fig 1:**
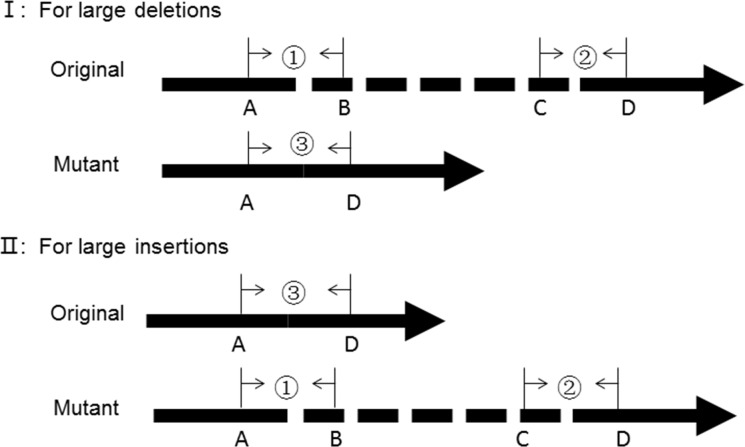
Schematic diagram of primer design for large indels. ①, ②, ③ were three pair primers designed for each large indel, and yield three amplicons AP1, AP2, AP3 respectively. BC was the inserted or deleted sequence.

Cholestasis was defined as direct bilirubin (DB) >20% of the total bilirubin (TB) if TB >5 mg/dL or DB >1 mg/dL if TB <5 mg/dL [10]. A custom AmpliSeq panel was designed to cover the target regions, including the coding DNA sequence and at least 5bp of flanking intronic regions, of 61 genes associated with cholestasis (S1 Table). Among the 61 genes, 25 were known intrahepatic cholestasis disease-causing genes [14–15]. The remaining 36 included potential candidate genes and genes for differential diagnoses. In addition, 25 related known large indels in these genes were also included in this panel (S2 Table). Three pair primers were designed for each large indel, and yield three amplicons (AP1, AP2 and AP3) covering the original allele and mutant allele (Fig 1). An internal program was encoded to detect the three amplicons. The implication of these amplicons was summarized in Table 1.

**Table 1:** The implication of amplicons for large indels detection.

AP1	AP2	AP3	Deletion	Insertion
**+**	**+**	**+**	Heterozygous	Heterozygous
**+**	**+**	**-**	Normal	Homozygous
**-**	**-**	**+**	Homozygous	Normal

AP1, AP2, AP3, three amplicons yield from primers designed for each large indel

### Subjects and Experiment Design

At validation phase, 54 cholestatic patients, who had received a clear genetic diagnosis by Sanger sequencing, were re-evaluated using this panel. Among them, 5 patients harbored large insertions in *SLC25A13*, and were diagnosed with neonatal intrahepatic cholestasis caused by citrin deficiency (NICCD). Both panel sequencing data and Sanger sequencing results of the 54 patients were used to optimize parameter settings of the data analysis protocol that helped to retain true positives and filter false positives effectively.

At implement phase, 141 consecutive patients with intrahepatic cholestasis were evaluated from April 2015 to November 2015. The patients were referred to the Center for Pediatric Liver Disease of the Children’s Hospital of Fudan University and the Department of Pediatrics, Jinshan Hospital of Fudan University. Following a reported extensive workup [16–17], other causes were excluded, including infections, drug-induced, metabolic and surgical causes. Cytomegalovirus (CMV) infection was considered if serum immunoglobulin M (IgM) or pp65 antigenemia or urinary CMV-DNA was positive [18]. CMV infection was not excluded for its high prevalence in Chinese infants [19]. The 141 patients included 92 boys and 49 girls. The age ranged from 1 month to 17 years old when panel sequencing was ordered.

This study was approved by the ethics committee of Jinshan Hospital of Fudan University and Children’s Hospital of Fudan University. Written informed consent was obtained from guardian/their parents. Patients’ information was de-identified prior to analysis.

### Library Construction, Enrichment and Sequencing

Library construction, enrichment and sequencing were performed according to the manufacturer’s instructions (Life Technologies, USA). Briefly, targets were amplified by a multiple polymerase chain reaction assay, and ligated to Ion Xpress barcode adapters. Then, libraries were purified and normalized to ~100pM. Template-positive Ion Sphere Particles were prepared and enriched. 8 samples were loaded per 316 v2 chip. Sequencing was performed on the Ion Torrent Personal Genome machine (Ion PGM) system.

### Data Analysis and Variations Classification

Torrent Suite, the Ion Torrent platform-specific pipeline software, was used to process raw data. Human genome reference sequence (hg19) was used for the reference. Variation calling was performed using settings [Homopolymer Indel Balance ≤0.5, and Frequency ≤50%] by NextGene software (version 2.3.3). For large indels test, panel sequencing data were further analyzed using the internal program encoded for known large indels test.

**Fig 2:**
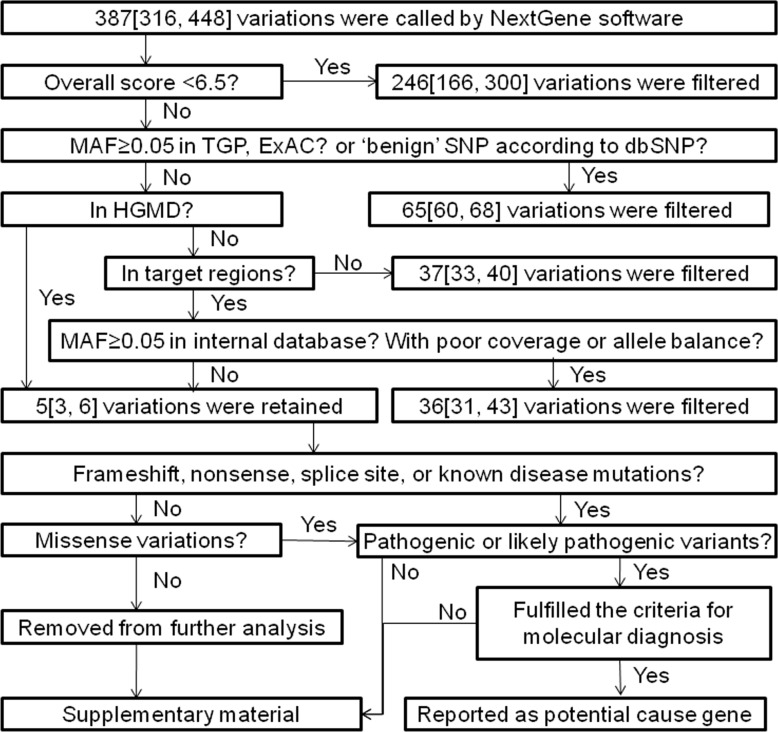
The optimized protocol for panel sequencing data analysis. The data from 195 patients were presented as median [P25, P75]. MAF, minor allele frequency; TGP, 1000 Genomes Project; ExAC, Exome Aggregation Consortium; dbSNP, Single Nucleotide Polymorphism database; HGMD, Human Gene Mutation Database.

Variations with suboptimal overall scores (OS) were filtered as false positives (Fig 2). Variations with minor allele frequency (MAF) ≥0.05 according to 1000 Genomes Project (TGP) or Exome Aggregation Consortium (ExAC) and benign single nucleotide polymorphism (SNP) according to dbSNP were considered benign and filtered [20]. Of the remaining variations, mutations in Human Gene Mutation Database (HGMD) were retained [21], while variations beyond 5bp from exon boundaries, and variations with MAF ≥0.05 in internal database or poor coverage (<20x) or poor allele balance were discarded.

Of the retained variations, frameshift, nonsense, canonical splice site variants, previous reported mutations and missense variations were analyzed further, while synonymous variations and variations beyond the canonical splice site were removed from further analysis. The prediction of missense variations was performed by *in silico* predictors (Polyphen 2, MutationTaster and SIFT). The pathogenicity was assessed according to the standards and guidelines for interpretation of sequence variants [20].

### Sanger Sequencing and Electrophoresis

Pathogenic and likely pathogenic variants of interest were confirmed by directly sequencing the affected exons from the patients and the parents using Sanger sequencing. Primer sequences and PCR conditions were available on request. Purified PCR products were directly sequenced on an ABI Prism 3500 Genetic Analyzer. Large indels were amplified by long and accurate-PCR (LA-PCR). LA-PCR products were confirmed by electrophoresis.

### Statistical Analysis

Statistic analysis was done using SPSS version 17.0 software (University of Chicago, Chicago, IL, United States). Data were expressed as mean±SD for normality, or median [P25, P75] for non-normality. Comparisons of two means or two medians were done by using two independent samples t-test or nonparametric Mann-Whitney test respectively. *P*<0.05 was considered significant.

## Results

### Performance of Ion PGM Sequencing

A total of 1278 pair primers were designed to cover the target regions of the 61 cholestasis associated genes, including 886 coding exons. The numbers of total reads and mapped reads, and the average depth of coverage were similar between patients at validation phase and at implementation phase (*P* >0.05, Table 2). Few exons were found to have bases with poor coverage (<20x) in patients at implementation phase, and the range was 13~41 (1.5%~4.6% of the 886 exons) while it was 23~43 (2.6%~4.9% of the 886 exons) for patients at validation phase.

**Table 2:** The performance of Ion PGM sequencing.

	At validation phase(N = 54)	At implementation phase(N = 141)
Total reads	36.5k±7.1k	39.0k±7.2k
Mapped reads	35.4k±6.7k	37.6k±7.0k
Average depth of coverage	277.2±52.6	294.2±54.5
Exons with poor coverage (<20x)	32.0 [29.0, 35.0]	28.0 [23.0, 32.0] *
Total called variations	417.0±80.3	366.4±103.3 *
Variations with OS ≥6.5	142.0 [136.0, 156.0]	139.5 [133.0, 151.0]
Retained variations	5.0 [4.0, 7.0]	4.0 [3.0, 5.0] *
Mutations per sample	2.0 [1.0, 2.0]	0.0 [0.0, 1.0] *

* Patients at validation phrase vs. patients at implement phrase, *P* <0.05

### Validation of Detection Efficiency

In the target regions of the 54 patients with known genetic diagnosis, Sanger sequencing identified 225 variations, including 11 distinct indels and 84 different substitutions. Of them, 224 (99.6%) were detected by panel sequencing with one missing for low coverage (5×). Additional 420 variations were identified by panel sequencing in the same regions, but not detected by Sanger sequencing. These variations were regarded as false positives, and the majority (99.3%) was indels. Tree false substitutions were identified and had OS<6.5. The features of false and true positives were summarized in Table 3. Following a data analysis protocol (Fig 2), 99.8% of false positives could be filtered. Of the 86 retained variations, 85 (98.8%) were true positives. The remaining one false positive (1.2%) was 1bp deletion.

**Table 3:** Features of false and true positives.

	True positives	False positives
Total	224	420
Substitutions	213 (95.1%)	3 (0.7%)
Indels	11 (4.9%)	417 (99.3%)
Overall score (OS)		
OS ≥6.5	224 (100.0%)	36 (8.6%)
OS >12.0	217 (96.9%)	24 (5.7%)
Original/mutant allele ratio <2.5:1	224 (100.0%)	127 (30.2%)
Filtered variations ^§^	139 (62.1%)	419 (99.8%)
OS <6.5 (false positives)	0 (0.0%)	384 (91.4%)
Benign variations ^‡^	129 (57.6%)	0 (0.0%)
Discarded variations ^†^	10 (4.5%)	35 (8.3%)

^§^ Filtered variations: 558 variations detected by panel sequencing were filtered following the protocol shown in Fig 2.

^‡^ Benign variations were defined as variations with MAF ≥0.05 in TGP and ExAC, or variations classified as benign in dbSNP.

^†^ Discarded variations included variations beyond the target regions, and variations with MAF ≥0.05 in internal database or poor coverage or poor allele balance.

### Re-evaluation of Patients with Known Genetic Diagnosis

In the 54 patients with known genetic diagnosis, 74 pathogenic or likely pathogenic variants were detected by Sanger sequencing. Following the data analyzing protocol in Fig 2, about 5 (range: 1~11) variations were retained by panel sequencing per sample (Table 2). The retained variations contained all the 74 pathogenic or likely pathogenic variants detected by Sanger sequencing. Then, panel sequencing data were further analyzed using the internal program encoded for large indels test. Large insertions were correctly detected in the 5 NICCD patients (Table 4). Therefore, by combination of the two data analysis methods, all known short genetic variants (≤50bp) and large indels, were detected successfully.

**Table 4:** Information of large indels identified in 5 citrin deficiency patients.

Patient	Gene	Mutation	Zygosity	NextGene	In-program ^‡^
NO.1	*SLC25A13*	c.329-18_329-17ins6057bp	Homozygous	Not	Yes
NO.2	*SLC25A13*	c.1751-5_1751-4ins3kb	Homozygous	Not	Yes
NO.3	*SLC25A13*	c.1751-5_1751-4ins3kb	Heterozygous	Not	Yes
	*SLC25A13*	c.1638_1660dup	Heterozygous	Yes	Not
NO.4	*SLC25A13*	c.1751-5_1751-4ins3kb	Homozygous	Not	Yes
NO.5	*SLC25A13*	c.1402C>T	Heterozygous	Yes	Not
	*SLC25A13*	c.1751-5_1751-4ins3kb	Heterozygous	Not	Yes

^‡^ In-program, internal program encoded by our team to detect known large indels.

### Evaluation of Patients without a Previous Genetic Diagnosis

Panel sequencing data of the 141 patients with intrahepatic cholestasis were analyzed using the same protocol (Fig 2). About 4 (range: 0~11) variations were retained per sample (Table 2). A total of 127 retained variations, including 110 substitutions and 17 indels, were chosen to validate by Sanger sequencing. Among them, 123 (96.9%; 123/127) were confirmed to be true, including 110 substitutions and 13 indels. Fourteen filtered variations, including 1 substitution with OS<6.5, 1 deletion with MAF>0.05 in internal database and 11 indels with OS>6.5 (range: 7.1~18.3) but original/mutant allele ratios ≥2.5:1, were confirmed to be false. Additional variations were not identified in target regions of the 138 affected exons evaluated by Sanger sequencing.

Pathogenic or likely pathogenic variants were identified in 59 (41.8%; 59/141) patients. Twenty-nine patients obtained a potential genetic diagnosis (Table 5). Twenty-three patients were diagnosed with autosomal recessive (AR) disorders and 6 had autosomal dominant (AD) disorders. Nine distinct genetic disorders were diagnosed, including 4 seen only once. For the 29 patients, a total of 36 different mutations were identified in causal genes. The 36 mutations included 2 deletions, 2 insertions, 5 nonsense, 7 canonical splice sites and 20 missense mutations. Of the 20 missense mutations, 6 were novel and predicted to be damaging (S3 Table).

**Table 5:** The spectrum of genetic disorders diagnosed by panel sequencing.

Patient	Gene	Nucleotide change	Amino acid change
1	*SLC25A13*	c.1177+1G>A/c.1177+1G>A	-/-
2	*SLC25A13*	c.1095delT/c.1157G>T	p. F365LfsX43/p.G386V
3	*SLC25A13*	c.851_854del/c.851_854del	p.M285PfsX2 /p.M285PfsX2
4	*SLC25A13*	c.851_854del/c.851_854del	p.M285PfsX2 /p.M285PfsX2
5	*SLC25A13*	c.851_854del/c.851_854del	p.M285PfsX2 /p.M285PfsX2
6	*SLC25A13*	c.851_854del/c.851_854del	p.M285PfsX2 /p.M285PfsX2
7	*SLC25A13*	c.851_854del/c.851_854del	p.M285PfsX2 /p.M285PfsX2
8	*SLC25A13*	c.851_854del/c.851_854del	p.M285PfsX2 /p.M285PfsX2
9	*ABCC2*	c.2302C>T/**c.4024T>C**	p.R768W/p.S1342P
10	*ABCC2*	**c.632+2_632+5del**/**c.4238_4239dup**	-/p.H1414LfsX18
11	*ABCC2*	c.3825C>G/c.4146+1G>T	p.Y1275X/-
12	*ABCC2*	**c.2366C>T**/**c.2366C>T**	p.S789F/p.S789F
13	*ABCC2*	**c.1963C>T**/**c.2153A>G**	p.R655X/p.N718S
14	*ABCC2*	**c.1281T>G**/**c.4025C>A**	p.D427E/p.S1342Y
15	*ABCC2*	**c.2224G>A**/**c.4025C>A**	p.D742N/p.S1342Y
16	*JAG1*	c.133G>T/-	p.V45L/-
17	*JAG1*	c.133G>T/-	p.V45L/-
18	*JAG1*	c.133G>T/-	p.V45L/-
19	*JAG1*	c.463G>C/-	p.A155P/-
20	*JAG1*	c.2698C>T/-	p.R900X/-
21	*ABCB11*	c.1550G>A/c.908+1G>T	p.R517H/-
22	*ABCB11*	c.3691C>T/c.872T>C	p.R1231W/p.V291A
23	*ABCB11*	**c.2197C>T**/**c.1489C>T**	p.Q733X/p.Q497X
24	*CYP27A1*	c.379C>T/c.1263+1G>A	p.R127W/-
25	*CYP27A1*	c.379C>T/c.1214G>A	p.R127W/p.R405Q
27	*CFTR*	c.214G>A/c.650A>G+c.3406G>A	p.A72T/p.E217G+ p.A1136T
27	*GALT*	**c.377+2dup**/**c.377+2dup**	-/-
28	*NOTCH2*	**c.6027+1G>A/**-	-/-
29	*NPC1*	c.1421C>T/c.2728G>A	p.P474L/p.G910S

Novel mutations are shown in bold.

### Large Indels Test Facilitates Genetic diagnosis

Sequencing data of the 141 patients were analyzed using the internal program encoded for large indels test. Large insertions were identified in *SLC25A13* in two patients, and were confirmed to be true by LA-PCR. Consequently, two additional patients obtained a potential genetic diagnosis, and were diagnosed with NICCD (one with c.1640_1641ins23bp/IVS4ins6kb and the other with c.851_854del/IVS16ins3kb). Hence, the rate of positive genetic diagnosis was 22.0%. The top 4 common genetic disorders: NICCD (32.3%), Dubin-Johnson syndrome (DJS, 22.6%), Alagille syndrome (19.4%), PFIC2/BRIC2 (9.8%).

## Discussion

Multiple specific genetic defects can cause intrahepatic cholestasis. It is still challenging to assign a molecular diagnosis because the differential diagnoses are numerous [2]. To ease the process of diagnosis, we designed a multi-gene panel that contained 61 genes associated with cholestasis and 25 related known large indels. We demonstrated that this panel was very practical, and that the ability of large indels detection could further facilitate genetic diagnosis. Using this panel, we assigned a potential molecular diagnosis to 22.0% of patients with intrahepatic cholestasis.

Poor coverage was one of the important causes for missing true positives [2]. In this study, more than 95.0% of the coding exons had desired coverage. Hence, bases with poor coverage (<20x) were much less than 5.0%. This might account for that 99.6% of known variations were successfully detected. However, numerous false positives were also produced during sequencing. The majority was indels, only a few (0.7%) were substitutions. At validation phase, all false substitutions had OS <6.5, while all true substitutions had OS ≥6.5. At implementation phase, all retained substitutions with OS ≥6.5 were confirmed to be true, while filtered substitutions with OS <6.5 were confirmed to be false. Hence, we inferred that OS ≥6.5 was reliable for true substitutions. It was different from previous researches [22–23]. According to our data, 3.1% of true positives would be missed if OS >12.0 was set as a cut-off value. Furthermore, 8.6% of false indels also had OS ≥6.5, even >12.0. Therefore, the retained variations also contained a few false indels. To filter these false indels effectively, additional parameters were needed, i.e. original/mutant allele ratio, as described in this study.

Similar presentation, symptoms and management of patients with intrahepatic cholestasis hindered genetic diagnosis [3–4]. Multi-gene panel overcame the complexity of candidate gene approach, and had advantages in evaluating not only patients with atypical presentations, but also clinical diagnoses associated with multiple candidate genes, and patients lacking a genetic diagnosis despite extensive Sanger sequencing [10,20,24]. Using a multi-gene panel, 9 distinct genetic disorders were diagnosed in this study. 4 were seen only once, and most were first reported in Chinese child patients with intrahepatic cholestasis. This indicated that this panel was very powerful for the assignment of genetic diagnosis. However, we failed to include all causal genes of cholestasis in this panel, especial those reported recently [25].This might be one of the reasons for that the majority (78.0%) of the patients still lacked a genetic diagnosis.

Large indels were commonly identified in patients [13], but failed to be detected by using routine Sanger sequencing and routine multi-gene panel sequencing [2]. Multiplex ligation-dependent probe amplification (MLPA) analysis, genomic microarrays, and other CNV analysis tools based on routine next generation sequencing (NGS) data were used to identify large indels characterized by DNA copy number loss/gain [10,26–27]. However, some special large indels, i.e. a retrotransposal insertion IVS16ins3kb that the inserted sequence was an antisense strand of complementary DNA (cDNA) processed from *C6orf68* and a transposal insertion IVS4ins6kb in *SLC25A13* [6,28], could not be detected by routine CNV analysis. These large indels had high frequency in patients, and were identified in 26.0% of NICCD patents and accounted for 14.3% of total mutant alleles [29]. The detection of these large indels could facilitate the diagnosis and improve the diagnostic efficiency. However, other labor-intensive and cost-expensive molecular tools were needed to identify them [11]. Using our design, we could simultaneously test these special large indels in addition to ordinary variants in a multi-gene panel. Our data demonstrated that this design was very practical to detect known large indels with definite chromosomal location. However, this panel failed to enroll other known large indels without definite chromosomal location, and failed to identify novel large indels. By using this panel, 22.0% patients with intrahepatic cholestasis obtained a potential genetic diagnosis, including two harboring large insertions in *SLC25A13*. Large indels were only identified in NICCD patients, it might attribute to that large indels had high frequency in *SLC25A13* and that NICCD was common in Asian [10,29]. Therefore, we suggest that *SLC25A13* should be compulsory after *CFTR*, which causes the most common AR genetic disorder in white population [30], in every panel for children with intrahepatic cholestasis. This is the first report to our knowledge that using panel sequencing to test known large indels in addition to small sequencing changes at one assay without CNV analysis. Without a doubt, the design of this panel can be used for other panels that involve known large indels, especial those failed to be detected by CNV analysis.

## Conclusion

We specially designed a multi-gene panel that contained 61 genes associated with cholestasis and 25 related known large indels. We demonstrated that this panel was practical and powerful in evaluation of patients with intrahepatic cholestasis. Using this panel, we assigned a potential genetic diagnosis to 22.0% of patients with intrahepatic cholestasis, including two patients with large indels.

## Supporting Information

S1 STROBE Checklist STROBE Statement—checklist of items that should be included in reports of observational studies. (DOCX)

S1 Table 61 genes included in multi-gene panel. (DOC)

S2 Table 25 known gross mutations included in multi-gene panel. (DOC)

S3 Table Predicted Effects of Novel Missense Mutations. (DOC)
