# Supplementary material for: A Specially Designed Multi-Gene Panel Facilitates Genetic Diagnosis in Children with Intrahepatic Cholestasis: Simultaneous Test of Known Large Insertions/Deletions
Source: PLoS One. 2016 Oct 5;11(10):e0164058. doi: 10.1371/journal.pone.0164058 (PMC5051675; doi:10.1371/journal.pone.0164058)
Supplement: S1 STROBE Checklist — (DOCX) [file pone.0164058.s001.docx]

STROBE Statement—checklist of items that should be included in reports of observational studies

|  | Item No. | Recommendation | Page  No. | Relevant text from manuscript |
| --- | --- | --- | --- | --- |
| **Title and abstract** | 1 | (*a*) Indicate the study’s design with a commonly used term in the title or the abstract | 1 | The validation phase was performed using 54 patients with known genetic diagnosis, including 5 with gross mutations. At implement phase, 141 patients with idiopathic cholestasis were evaluated. |
|  |  | (*b*) Provide in the abstract an informative and balanced summary of what was done and what was found | 1 | reported the diagnostic yield in patients with idiopathic cholestasis |
| Introduction | | | |  |
| Background/rationale | 2 | Explain the scientific background and rationale for the investigation being reported | 2-3 | Quite often, several candidate genes have to be evaluated in clinic practice, because the differential diagnoses are numerous  large insertions/deletions can neither be detected by routine Sanger sequencing, nor by routine multi-gene panel sequencing, even by copy number variation (CNV) analysis. Furthermore, some gross mutations have high frequency in patients, and contribute substantively to disease burden |
| Objectives | 3 | State specific objectives, including any prespecified hypotheses | 3 | To facilitate genetic diagnosis, we specially designed a multi-gene panel |
| Methods | | | |  |
| Study design | 4 | Present key elements of study design early in the paper | 3 | 61 genes associated with cholestasis and 25 related known gross indels were included in this panel |
| Setting | 5 | Describe the setting, locations, and relevant dates, including periods of recruitment, exposure, follow-up, and data collection | 4 | Between January 2015 and November 2015, 195 patients were evaluated using this panel, including 54 patients with known genetic diagnosis and 141 unselected patients with idiopathic cholestasis. |
| Participants | 6 | (*a*) *Cohort study*—Give the eligibility criteria, and the sources and methods of selection of participants. Describe methods of follow-up  *Case-control study*—Give the eligibility criteria, and the sources and methods of case ascertainment and control selection. Give the rationale for the choice of cases and controls  *Cross-sectional study*—Give the eligibility criteria, and the sources and methods of selection of participants | 4 | patients were evaluated using this panel  Other causes were excluded |
|  |  | (*b*)*Cohort study*—For matched studies, give matching criteria and number of exposed and unexposed  *Case-control study*—For matched studies, give matching criteria and the number of controls per case | 3 | we first validated our system using 54 patients with known genetic diagnosis, then evaluated 141 unselected patients with idiopathic cholestasis using this panel |
| Variables | 7 | Clearly define all outcomes, exposures, predictors, potential confounders, and effect modifiers. Give diagnostic criteria, if applicable | 5 | Frameshift, nonsense, canonical splice site variants and previous reported mutations were considered pathogenic. Missense variations predicted to be damaging by in silico predictors were considered as likely pathogenic mutations |
| Data sources/measurement | 8* | For each variable of interest, give sources of data and details of methods of assessment (measurement). Describe comparability of assessment methods if there is more than one group | 3,5 | An internal program was encoded to detect the three amplicons.  Data Analysis and Variations Classification |
| Bias | 9 | Describe any efforts to address potential sources of bias | 4 | Patients’ information was de-identified prior to analysis. |
| Study size | 10 | Explain how the study size was arrived at | 3-4 | 195 patients were evaluated using this panel.  we first validated our system using 54 patients with known genetic diagnosis were used to validate our system; then we evaluated 141 unselected patients with idiopathic cholestasis using this panel |

Continued on next page

| Quantitative variables | 11 | Explain how quantitative variables were handled in the analyses. If applicable, describe which groupings were chosen and why | 5 | Data Analysis and Variations Classification |
| --- | --- | --- | --- | --- |
| Statistical methods | 12 | (*a*) Describe all statistical methods, including those used to control for confounding | 6 | Chi-square test was used to test the difference between two ratios. P<0.05 was considered significant. |
|  |  | (*b*) Describe any methods used to examine subgroups and interactions | N/A | no |
|  |  | (*c*) Explain how missing data were addressed | N/A | no |
|  |  | (*d*) *Cohort study*—If applicable, explain how loss to follow-up was addressed  *Case-control study*—If applicable, explain how matching of cases and controls was addressed  *Cross-sectional study*—If applicable, describe analytical methods taking account of sampling strategy | N/A | no |
|  |  | (*e*) Describe any sensitivity analyses | N/A | no |
| Results | | | | |
| Participants | 13* | (a) Report numbers of individuals at each stage of study—eg numbers potentially eligible, examined for eligibility, confirmed eligible, included in the study, completing follow-up, and analysed | 6-8 | Validation of Detection Efficiency  Re-evaluation of Patients with Known Genetic Diagnosis  Evaluation of Patients with Idiopathic Cholestasis |
|  |  | (b) Give reasons for non-participation at each stage | N/A | no |
|  |  | (c) Consider use of a flow diagram | N/A | no |
| Descriptive data | 14* | (a) Give characteristics of study participants (eg demographic, clinical, social) and information on exposures and potential confounders | 8-9 | Table 4. The spectrum of genetic disorders diagnosed by panel sequencing |
|  |  | (b) Indicate number of participants with missing data for each variable of interest | N/A | no |
|  |  | (c) *Cohort study*—Summarise follow-up time (eg, average and total amount) | N/A | no |
| Outcome data | 15* | *Cohort study*—Report numbers of outcome events or summary measures over time | 8-9 | Table 4. The spectrum of genetic disorders diagnosed by panel sequencing  Large insertions were identified in SLC25A13 in two patients |
|  |  | *Case-control study—*Report numbers in each exposure category, or summary measures of exposure | N/A | no |
|  |  | *Cross-sectional study—*Report numbers of outcome events or summary measures | N/A | no |
| Main results | 16 | (*a*) Give unadjusted estimates and, if applicable, confounder-adjusted estimates and their precision (eg, 95% confidence interval). Make clear which confounders were adjusted for and why they were included | 6-7 | Table 2. Features of false and true positives |
|  |  | (*b*) Report category boundaries when continuous variables were categorized | N/A | no |
|  |  | (*c*) If relevant, consider translating estimates of relative risk into absolute risk for a meaningful time period | N/A | no |

Continued on next page

| Other analyses | 17 | Report other analyses done—eg analyses of subgroups and interactions, and sensitivity analyses | 6-8 | by combination of the two analysis methods, all known substitutions, small indels, and gross mutations, were detected successfully. |
| --- | --- | --- | --- | --- |
| Discussion | | | | |
| Key results | 18 | Summarise key results with reference to study objectives | 9 | We demonstrated that this panel was very practical, and that the ability of gross mutation detection could further facilitate genetic diagnosis. Using this panel, we assigned a potential molecular diagnosis to 24.1% of patients with idiopathic cholestasis. |
| Limitations | 19 | Discuss limitations of the study, taking into account sources of potential bias or imprecision. Discuss both direction and magnitude of any potential bias | 11 | The technical limitations |
| Interpretation | 20 | Give a cautious overall interpretation of results considering objectives, limitations, multiplicity of analyses, results from similar studies, and other relevant evidence |  | gross mutation failed to be detected by routine Sanger analysis and Multi-gene panel sequencing, even by CNV analysis. Using a special design, we could simultaneously test known gross mutations in addition to ordinary variants in a multi-gene panel, and 24.1% patients with idiopathic cholestasis obtained a genetic diagnosis, including two harboring large insertions in SLC25A13. |
| Generalisability | 21 | Discuss the generalisability (external validity) of the study results | 11 | the design of this panel can be used for other panels that involve not only small sequencing changes, but also known gross insertion/deletions. |
| Other information | |  | | |
| Funding | 22 | Give the source of funding and the role of the funders for the present study and, if applicable, for the original study on which the present article is based |  |  |

*Give information separately for cases and controls in case-control studies and, if applicable, for exposed and unexposed groups in cohort and cross-sectional studies.

**Note:** An Explanation and Elaboration article discusses each checklist item and gives methodological background and published examples of transparent reporting. The STROBE checklist is best used in conjunction with this article (freely available on the Web sites of PLoS Medicine at http://www.plosmedicine.org/, Annals of Internal Medicine at http://www.annals.org/, and Epidemiology at http://www.epidem.com/). Information on the STROBE Initiative is available at www.strobe-statement.org.
