# Supplementary material for: A Specially Designed Multi-Gene Panel Facilitates Genetic Diagnosis in Children with Intrahepatic Cholestasis: Simultaneous Test of Known Large Insertions/Deletions
Source: PLoS One. 2016 Oct 5;11(10):e0164058. doi: 10.1371/journal.pone.0164058 (PMC5051675; doi:10.1371/journal.pone.0164058)
Supplement: S1 Table — (DOC) [file pone.0164058.s002.doc]

**S1 Table. 61 genes included in multi-gene panel**

| ***ABCB11*** (NM_003742.2) | ***ABCB4*** (NM_000443.3) | ***ABCC2*** (NM_000392.3) |
| --- | --- | --- |
| *ADK* (NM_006721.3) | ***AKR1D1*** (NM_005989.3) | *ALDOA* (NM_000034.2) |
| *ALDOB* (NM_000035.3) | ***AMACR*** (NM_014324.5) | *ANKS6* (NM_173551.3) |
| *AP1S1* (NM_001283.3) | ***ATP8B1*** (NM_005603.4) | ***BAAT*** (NM_001701.3) |
| *BCS1L* (NM_004328.4) | ***CFTR*** (NM_000492.3) | ***CIRH1A*** (NM_032830.2) |
| ***CLDN1*** (NM_021101.4) | ***CYP27A1*** (NM_000784.3) | ***CYP7B1*** (NM_004820.3) |
| *DGUOK* (NM_080916.1) | *DHCR7* (NM_001360.2) | *EARS2* (NM_001083614.1) |
| *ETFA* (NM_000126.3) | *ETFB* (NM_001985.2) | *ETFDH* (NM_004453.2) |
| ***FAH*** (NM_000137.2) | *FH* (NM_000143.3) | *GALE* (NM_000403.3) |
| ***GALT*** (NM_000155.2) | *GBA* (NM_001005741.2) | *GFM1* (NM_024996.5) |
| *HESX1* (NM_003865.2) | *HSD17B4* (NM_000414.3) | ***HSD3B7*** (NM_025193.3) |
| *IFT140* (NM_014714.3) | *IFT43* (NM_052873.2) | *INSR* (NM_000208.2) |
| ***JAG1*** (NM_000214.2) | *KRT18* (NM_000224.2) | *KRT8* (NM_002273.3) |
| ***LIPA*** (NM_000235.2) | *MPV17* (NM_002437.4) | ***NOTCH2*** (NM_024408.3) |
| ***NPC1*** (NM_000271.4) | ***NPC2*** (NM_006432.3) | *NPHP3* (NM_153240.4) |
| *PEX1* (NM_000466.2) | *PEX5* (NM_001131025.1) | *POU1F1* (NM_000306.2) |
| *SC5DL* (NM_001024956.2) | ***SERPINA1*** (NM_000295.4) | *SLC17A5* (NM_012434.4) |
| ***SLC25A13*** (NM_014251.2) | *SLC27A5* (NM_012254.2) | *SPTLC2* (NM_004863.3) |
| ***TJP2*** (NM_004817.3) | *TRMU* (NM_018006.4) | *TTC37* (NM_014639.3) |
| *UBR1* (NM_174916.2) | *UGT1A1* (NM_000463.2) | ***VIPAS39*** (NM_022067.3) |
| ***VPS33B*** (NM_018668.3) |  |  |

known intrahepatic cholestasis disease-causing genes are shown in bold.
