# Supplementary material for: A Specially Designed Multi-Gene Panel Facilitates Genetic Diagnosis in Children with Intrahepatic Cholestasis: Simultaneous Test of Known Large Insertions/Deletions
Source: PLoS One. 2016 Oct 5;11(10):e0164058. doi: 10.1371/journal.pone.0164058 (PMC5051675; doi:10.1371/journal.pone.0164058)
Supplement: S2 Table — (DOC) [file pone.0164058.s003.doc]

**S2 Table. 25 known gross mutations included in multi-**gene panel

| Gene | Gross deletion/insertion mutation |
| --- | --- |
| *ATP8B1* | c.2708-552_2724del569bp |
| *ABCB11* | c.1308+1048_2076-670del10.5kb |
| *JAG1* | c.331_387+118del175bp |
| *SLC25A13* | c.70-862_c.212+3527del4532bp |
| *SLC25A13* | c.1665_1842-32del516 |
| *SLC25A13* | c.1751-5_1751-4ins3kb |
| *SLC25A13* | c.329-18_329-17ins6057bp |
| *ABCC2* | c.633-275_867+498del1008bp |
| *ALDOB* | c.541-154_799+233del1573bp |
| *ALDOB* | c.325-664_476del1642bp |
| *CYP27A1* | c.1263+81_*1596+1420del1911bp |
| *FAH* | c.960+1130_*1260+10539del18036bp |
| *FAH* | c.1-3951_81+3887del7919bp |
| *GALT* | c.377+53_1059+87del1894bp |
| *UGT1A1* | c.1-2350_864+1377del4591bp |
| *HSD17B4* | c.230_289del1305bp |
| *CFTR* | c.54-5490_273+10250del20630bp |
| *CFTR* | c.54-1161_164+1603del2875bp |
| *CFTR* | c.3964-78_4242+577del1532bp |
| *CFTR* | c.2988+1173_3468+2111del8899bp |
| *CFTR* | c.2620-674_3367+198del9855bp |
| *CFTR* | c.2908+1085_3367+260del7202bp |
| *CFTR* | c.53+9713_1209+2669del54918bp |
