# Supplementary material for: A Specially Designed Multi-Gene Panel Facilitates Genetic Diagnosis in Children with Intrahepatic Cholestasis: Simultaneous Test of Known Large Insertions/Deletions
Source: PLoS One. 2016 Oct 5;11(10):e0164058. doi: 10.1371/journal.pone.0164058 (PMC5051675; doi:10.1371/journal.pone.0164058)
Supplement: S3 Table — (DOC) [file pone.0164058.s004.doc]

S3 Table. Predicted Effects of Novel Missense Mutations

| Gene | Mutation | Mutation Taster | | Polyphen-2 | | SIFT | |
| --- | --- | --- | --- | --- | --- | --- | --- |
|  |  | Prediction | *P* Value | Prediction | Score | Prediction | Score |
| *ABCC2* | c.4024T>C, p.S1342P | disease causing | 0.999 | Probably damaging | 1.000 | Damaging | 0.001 |
| *ABCC2* | c.2366C>T, p.S789F | disease causing | 0.999 | Probably damaging | 1.000 | Damaging | 0.000 |
| *ABCC2* | c.2153A>G, p.N718S | disease causing | 0.999 | Probably damaging | 1.000 | Damaging | 0.017 |
| *ABCC2* | c.1281T>G, p.D427E | disease causing | 0.999 | Probably damaging | 1.000 | Damaging | 0.000 |
| *ABCC2* | c.4025C>A, p.S1342Y | disease causing | 0.999 | Probably damaging | 1.000 | Damaging | 0.000 |
| *ABCC2* | c.2224G>A, p.D742N | disease causing | 0.999 | Probably damaging | 1.000 | Damaging | 0.001 |
